# Patterns of use and impact of standardised MedDRA query analyses on the safety evaluation and review of new drug and biologics license applications

**DOI:** 10.1371/journal.pone.0178104

**Published:** 2017-06-01

**Authors:** Lin-Chau Chang, Riaz Mahmood, Samina Qureshi, Christopher D. Breder

**Affiliations:** 1 Master of Science in Regulatory Science Program, Advanced Academic Programs, Krieger School of Arts and Sciences, Johns Hopkins University, Rockville, Maryland, United States of America; 2 School of Osteopathic Medicine, Campbell University, Lillington, North Carolina, United States of America; 3 Healthcare/Pharma, PSI International Inc., Fairfax, Virginia, United States of America; 4 Division of Neurology Products (DNP), Office of Drug Evaluation 1 (ODE1), Office of New Drugs (OND), Center for Drug Evaluation and Research (CDER), United States Food and Drug Administration (USFDA), Silver Spring, Maryland, United States of America; Universita degli Studi di Firenze, ITALY

## Abstract

**Purpose:**

Standardised MedDRA Queries (SMQs) have been developed since the early 2000’s and used by academia, industry, public health, and government sectors for detecting safety signals in adverse event safety databases. The purpose of the present study is to characterize how SMQs are used and the impact in safety analyses for New Drug Application (NDA) and Biologics License Application (BLA) submissions to the United States Food and Drug Administration (USFDA).

**Methods:**

We used the PharmaPendium database to capture SMQ use in Summary Basis of Approvals (SBoAs) of drugs and biologics approved by the USFDA. Characteristics of the drugs and the SMQ use were employed to evaluate the role of SMQ safety analyses in regulatory decisions and the veracity of signals they revealed.

**Results:**

A comprehensive search of the SBoAs yielded 184 regulatory submissions approved from 2006 to 2015. Search strategies more frequently utilized restrictive searches with “narrow terms” to enhance specificity over strategies using “broad terms” to increase sensitivity, while some involved modification of search terms. A majority (59%) of 1290 searches used descriptive statistics, however inferential statistics were utilized in 35% of them. Commentary from reviewers and supervisory staff suggested that a small, yet notable percentage (18%) of 1290 searches supported regulatory decisions. The searches with regulatory impact were found in 73 submissions (40% of the submissions investigated). Most searches (75% of 227 searches) with regulatory implications described how the searches were confirmed, indicating prudence in the decision-making process.

**Conclusions:**

SMQs have an increasing role in the presentation and review of safety analysis for NDAs/BLAs and their regulatory reviews. This study suggests that SMQs are best used for screening process, with descriptive statistics, description of SMQ modifications, and systematic verification of cases which is crucial for drawing regulatory conclusions.

## Introduction

The approval of a new drug is based on a balance of the substantial evidence of efficacy and its safety [1, 2]. For safety evaluations, investigations of clinical trial databases, often containing thousands of adverse events (AEs), must be critically reviewed and analyzed [2]. Safety signal identification is critically reliant on coding AEs into standardised terminology [3–5]. The Medical Dictionary for Regulatory Activities (MedDRA), a five-tiered hierarchical and multiaxial system of medical terminology, facilitates retrieval and presentation from different data sources [5, 6]. Standardised MedDRA Queries (SMQs) are groupings of about 50 to 200 MedDRA terms, generally at the Preferred Term (PT) level that defines specific medical conditions, such as hepatic failure or anaphylactic reactions [5–11]. Their development began in the early 2000’s and is currently maintained by the Council for International Organizations of Medical Sciences (CIOMS) Working Group who continually optimizes the list and the content of SMQs [5, 7]. Subsequent use by academia, industry, public health, and government sectors for detecting safety signals in AE safety databases has increased dramatically [5, 7].

SMQs are similar to a net that enables retrieval of cases of interest from MedDRA-coded databases [5, 7, 11]. The SMQs are designed with various features, such as narrow and broad scope terms, hierarchical structure, and for a few of them, an algorithmic option. Users of SMQs may apply these different features in diverse situations to meet their analytical needs [5, 7, 10, 11]. These features have greatly enhanced the performance of SMQ searches despite each SMQ not having all of these options [5, 7, 10, 11]. Irrelevant cases might still be retrieved despite the sophisticated design, which is a consequence more often associated with broad searches [11]. Therefore, case verification serves as an essential step in distinguishing signal from noise [11].

As development and use of SMQs have become increasingly important for safety analyses, a comprehensive evaluation into how SMQs are used and their impact is imperative. The objectives of this study are to characterize patterns in SMQ use and impact in the generation of regulatory decisions in the review of NDA and BLA submissions to the United States Food and Drug Administration (USFDA). Although the performance of SMQs has been discussed in previous research [12–18], the present study is the first one, to the best of our knowledge, to systematically and comprehensively encompass our objectives.

## Materials and methods

### Data sources

We used PharmaPendium^®^ (Elsevier) to search (last accessed on July 27, 2016) for USFDA Summary Basis of Approvals (SBoAs) in which use of SMQs was described. Publicly available reviews from each identified submission were extracted from the Drugs@FDA database of USFDA approved drug products [19]. Documents from different review disciplines were collated into a single PDF document and optical character recognition (Adobe Acrobat Pro DC; 2015 Version (2015006.030201)) was performed to make each file searchable.

### Features of SMQ use and analysis

A database was constructed in Microsoft EXCEL^®^ 2013 for information retrieved from SBoAs, which was established by the authors and verified by the corresponding author. The data included “demographic” information related to the drug and marketing application and “content” information related to the SMQ and how it was used (Table 1). Descriptive statistics were generated with JMP^®^ version 12.1.0 (SAS Institute Inc.). Percentages have been rounded to the nearest integer. Each supplement was considered as an independent submission.

**Table 1:** Demographic and content variables collected from the review documents.

Demographic variables related to the drug and marketing application	Content information related to the SMQ and how it was used
• Application number • Supplement number • Type (New Drug Application (NDA) or Biologics License Application (BLA)) • Trade name • Name(s) of the active ingredient(s) • Indication • Molecular class/Anatomical • Therapeutic Chemical classification system, 4^th^ level [20] • Therapeutic class/Anatomical • Therapeutic Chemical classification system, 2^nd^ level [20] • Year of the approval • Name of the applicant • Principal review division • Type of review document • Role of reviewer mentioning the SMQ use or including in their review	• Current SMQ names (current name in the Introductory Guide for Standardised MedDRA Queries (SMQs) Version 18.0) [7] • Whether cases were detected from the group with the administration of the study drug • Search option(s) applied (broad/narrow/algorithmic) • Modification of search terms • Database(s) searched • Search program(s) used • Initiation of SMQ searches • Verification of search results • Comparison of search results with other evidence • Whether the timing of the events was checked for algorithmic searches • Reporting methods (descriptive/inferential statistics) • Management concurrence • Summary of finding • Regulatory implications

SMQs, Standardised MedDRA Queries; MedDRA, Medical Dictionary for Regulatory Activities.

## Results

### Basic characteristics of the submissions, therapeutics analyzed, and SMQs used

**Fig 1:**
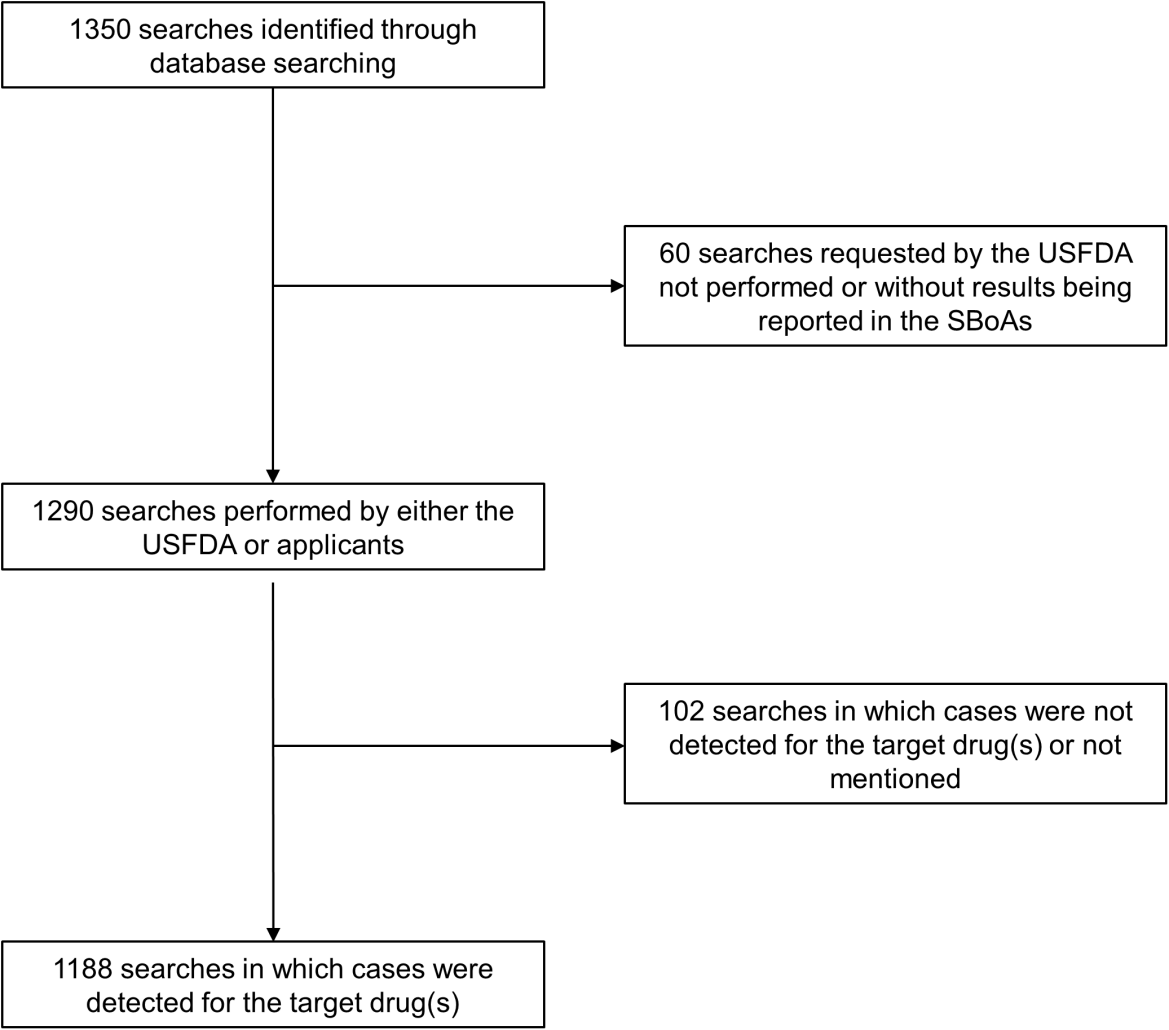
The diagram of the numbers of Standardised MedDRA Query (SMQ) searches investigated. SBoAs, Summary Basis of Approvals; USFDA, United States Food and Drug Administration.

**Fig 2:**
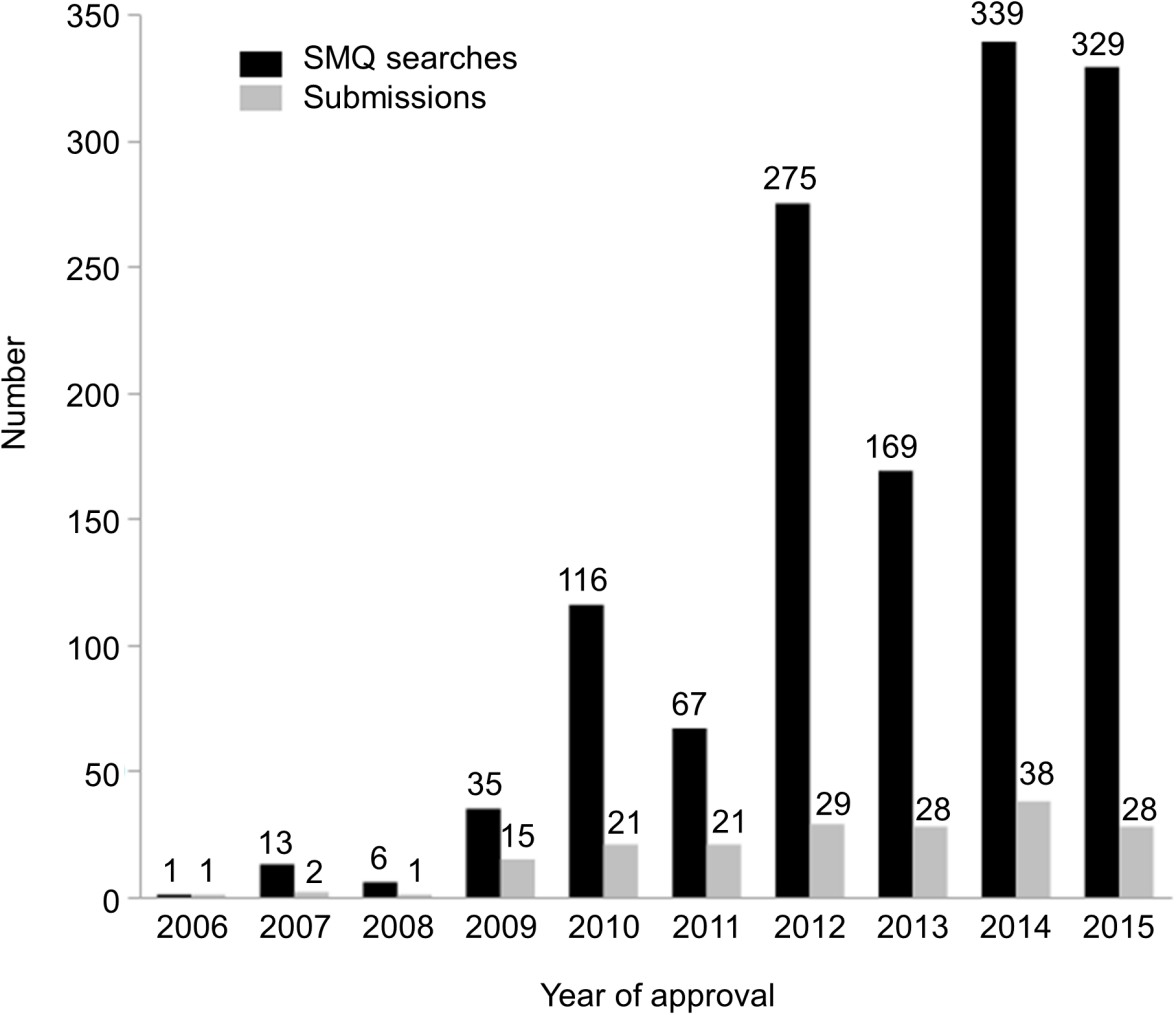
The numbers of Standardised MedDRA Query (SMQ) searches and submissions per year of approval.

In the present study, 184 submissions approved from 2006 to 2015 mentioned the use of SMQs. A total of 1350 SMQ investigations (Fig 1), including requests for searches not performed, were studied. The distribution of searches and submissions containing SMQ searches by year of the NDA/BLA approval is displayed in Fig 2. The majority (80%) of the 184 submissions analyzed were NDAs, though 20% of the submissions were BLAs. Most (157 (85%)) submissions were for the original NDA or BLA, with only 15% being post-approval supplements.

The top three therapeutic classes of therapeutics investigated with SMQs as represented by the second level of the Anatomical Therapeutic Chemical (ATC) classification system [20] were antineoplastic agents (32 (17%)), drugs used in diabetes (16 (9%)), and immunosuppressants (13 (7%)). The top three molecular classes represented by the fourth level of the ATC classification system were monoclonal antibodies (11 (6%)), protein kinase inhibitors (11 (6%)), and other blood glucose lowering drugs, excl. insulins (7 (4%)).

**Fig 3:**
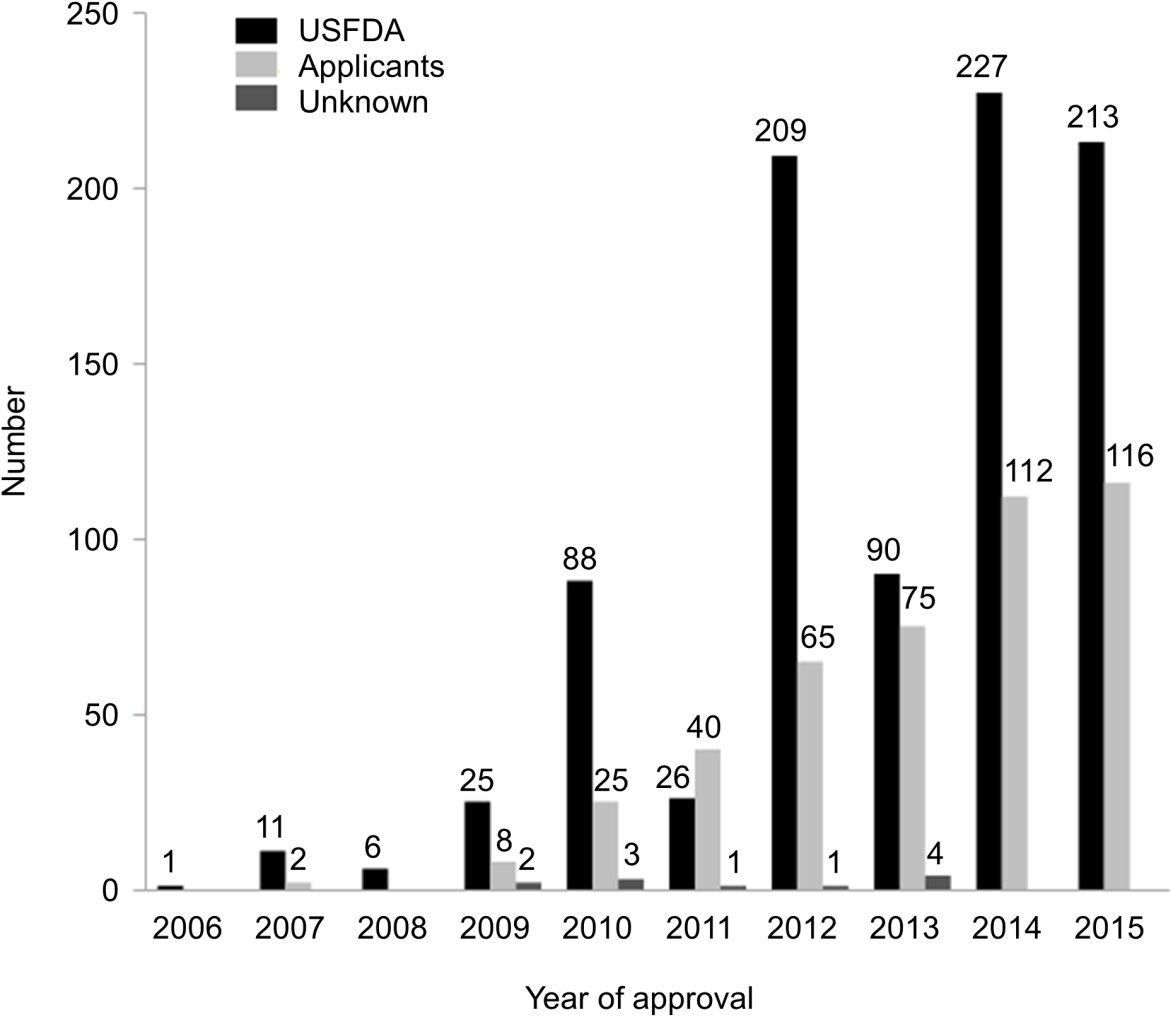
The numbers of SMQ searches initiated by the USFDA and applicants per year of approval. SMQ, Standardised MedDRA Query; USFDA, United States Food and Drug Administration.

The distribution of searches initiated by the USFDA and the applicants by year of the NDA/BLA approval is displayed in Fig 3. Most of the 1350 searches (896 (66%)) were initiated by the USFDA. For the 192 individual SMQ searches requested by the USFDA, the most frequent time of request was at pre-NDA/pre-BLA meetings where 103 (54%) searches were requested. A total of 60 (31%) searches requested by the USFDA were either not performed or the results were not reported in the SBoAs. 55 of these requests were found at pre-NDA/pre-BLA meeting minutes or information requests, or end-of-phase 2 meeting minutes. The primary medical/safety reviewers were the primary group describing SMQ searches. Nearly 90% of the searches were discovered in their reviews, while some search findings or requests were discussed in multiple review documents, including about 23% of cases that were discussed in reviews by supervisory staff. Additionally, the use of MedDRA-based Adverse Event Diagnostics (MAED) [21], a tool capable of searching AE databases for all SMQs, was mentioned in 425 searches, most of which (389 searches (92%)) were conducted by the USFDA. The frequency of use of MAED was generally increased over the years. MAED has been applied in 7 searches on average per year from 2009 to 2011 but in 101 searches on average per year from 2012 to 2015.

### Most common safety concerns evaluated by SMQ searches

85 first-level SMQs and 86 secondary or greater-level sub-SMQs were detected in these cases. Among the 1350 searches, the top five most frequently searched SMQs consisted of *Hepatic disorders (SMQ)* (166 (12%)), *Cardiac arrhythmias (SMQ)* (100 (7%)), *Ischaemic heart disease (SMQ)* (62 (5%)), *Central nervous system vascular disorders (SMQ)* (61 (5%)), and *Severe cutaneous adverse reactions (SMQ)* (51 (4%)), if each sub-SMQ was incorporated into the respective first-level SMQs. These findings demonstrated the most common safety concerns, including hepatic toxicity, cardiac toxicity, central nervous system toxicity, and hypersensitivity during evaluation of marketing applications using SMQs. Moreover, the distribution of the top three most frequently searched SMQs for the top three therapeutic classes represented by the second level of the ATC classification system is shown in Table 2 reflecting therapeutic class-specific considerations. The most frequently searched SMQs/sub-SMQs initiated by the USFDA was *Drug related hepatic disorders—comprehensive search (SMQ)* (41 searches (5%)), followed by *Severe cutaneous adverse reactions (SMQ)* (37 searches (4%)). In contrast, the most frequently searched SMQ/sub-SMQ initiated by the applicants was *Anaphylactic reaction (SMQ)* (24 searches (5%)), followed by *Cardiac failure (SMQ)* (19 searches (4%)), demonstrating the unique concerns from different perspectives. Interestingly, however, among the 55 searches described in the post-approval supplements, *Embolic and thrombotic events (SMQ)* (8 searches (15%)) became the most frequently-mentioned SMQs, implying different emphasis in the post-approval stage.

**Table 2:** Distribution of the top three most frequently searched SMQs for the top three therapeutic classes represented by the second level of the ATC classification system^a^^,^^b^.

Antineoplastic agents (462 searches)	Drugs used in diabetes (100 searches)	Immunosuppressants (77 searches)
*Hepatic disorders (SMQ)* (53 searches) (11%)	*Hepatic disorders (SMQ)* (19 searches) (19%)	*Cardiac arrhythmias (SMQ)* (10 searches) (13%)
*Cardiac arrhythmias (SMQ)* (31 searches) (7%)	*Angioedema (SMQ)* (9 searches) (9%)	*Hepatic disorders (SMQ)* (10 searches) (13%)
*Malignancies (SMQ)* (22 searches) (5%)	*Acute pancreatitis (SMQ)*; *Severe cutaneous adverse reactions (SMQ)* (8 searches) (8%)	*Malignancies (SMQ)* (6 searches) (8%)

SMQs, Standardised MedDRA Queries; MedDRA, Medical Dictionary for Regulatory Activities; ATC, Anatomical Therapeutic Chemical; sub-SMQs, subordinate SMQs.

^a^Since the ATC code was not available for the active ingredient(s) in 137 searches, these searches were not included, which would not affect the order of the top three therapeutic classes after manual designation.

^b^The sub-SMQs were incorporated into the respective first-level SMQs in this analysis.

### Balancing specificity and sensitivity

A search with narrow or broad scope terms could lead to different results emphasizing either specificity or sensitivity, while an algorithmic search utilizing the combinations of term categories is generally used in large databases to increase specificity. Although not all options are available for each SMQ, narrow searches were performed more frequently when both narrow and broad terms were available. Among 728 SMQ searches using first-level and non-hierarchical SMQs with both options, 39% of searches utilized only narrow terms, while 9% of them applied only broad terms. Fourteen percent of the searches compared the search results using both options while search options regarding the use of narrow or broad terms were not clearly specified in the rest of the searches. Although a potential 73 searches (5%) could have utilized algorithmic SMQs, only 21% of these searches reported applying the option. The relative timing of events in different term categories for algorithmic SMQs was reported in only 2 searches applying this search option.

Five percent of total searches involved modification of terms (addition of terms: 38 searches (53%); exclusion of terms: 31 searches (43%); addition and exclusion of terms: 3 searches (4%)) which, by convention, should not be referred to as SMQ searches due to their modification [7, 10, 11]. Among these 72 searches with modified search terms, more than 70% of the modification were initiated by the applicants. The SMQs (incorporating respective sub-SMQs, if applicable) most often modified were *Cardiac arrhythmias (SMQ)* (8 searches) and *Anaphylactic reaction (SMQ)* (7 searches).

### Confirmation of the safety signals identified by SMQ searches

Among 1188 searches in which cases were detected for the target drug(s), verification of cases retrieved was mentioned for 470 searches (40%). “Direct verification” was performed by the investigation of AEs by e.g., studying patient narratives, checking laboratory results or vital signs. Comparison of results with other evidence, such as the report on higher MedDRA levels of AEs was an alternative approach described for 518 searches (44%) in order to help substantiate the legitimacy of safety signals. For example, the search results with MedDRA High Level Terms were often analyzed in juxtaposition to SMQ searches to assess the similarity trends in the incidence of the two datasets. The utilization of at least one approach was described in 737 (62%) searches, while application of both confirmation methods was mentioned in 251 (21%) searches. The type of data used for verification of SMQ-positive cases outside of an examination of the AE PTs depended on respective safety issues. For example, *Drug related hepatic disorders—comprehensive search (SMQ)* was most often corroborated with clinical laboratory results. In contrast, *Torsade de pointes/QT prolongation (SMQ)* cases were generally verified with electrocardiogram interval data. The approaches used for the top five most frequently searched SMQs are displayed in Table 3.

**Table 3:** Confirmation approaches used for top five most frequently searched SMQs.

Top five most frequently searched SMQs^a^	Number of searches with direct verification of cases retrieved only (%)^b^	Number of searches with comparison of search results with other evidence^c^ only (%)^b^	Number of searches with the application of both approaches (%)^b^	Number of searches without mentioning of either approach (%)^b^	Total number of searches with cases detected for the drug(s) investigated (%)^b^
*Drug related hepatic disorders—comprehensive search (SMQ)*^d^	9 (28)	2 (6)	12 (38)	9 (28)	32 (100)
*Severe cutaneous adverse reactions (SMQ)*^d^	8 (25)	3 (9)	13 (41)	8 (25)	32 (100)
*Anaphylactic reaction (SMQ)*^d^	11 (31)	3 (9)	12 (34)	9 (26)	35 (100)
*Angioedema (SMQ)*^d^	10 (33)	5 (17)	9 (30)	6 (20)	30 (100)
*Torsade de pointes/QT prolongation (SMQ)*^d^	5 (17)	5 (17)	10 (34)	9 (31)	29 (100)

SMQs, Standardised MedDRA Queries; MedDRA, Medical Dictionary for Regulatory Activities.

^a^Based on total number of searches; the sub-SMQ was not incorporated into the respective first-level SMQs in this analysis.

^b^Based on number of searches with cases detected for the drug(s) investigated.

^c^Such as the comparison with the report on higher MedDRA levels of adverse events.

^d^Due to rounding, the percentages may not add to 100%.

### Reporting of findings

Among 1290 searches performed by either the USFDA or the applicants (excluding requests not performed), most results (763 searches (59%)) were reported using descriptive statistics. For 454 (35%) searches that included inferential statistics in their presentation of results (whether results were found or not), 39% reported a combination of the relative risk, the number of cases, and incidence, with or without a p-value; confidence intervals were reported in 98 (22%) searches. Among the 442 searches where cases were found for the target drug(s) and that used inferential statistics, direct verification of cases was mentioned for 97 (22%) searches.

### SMQ searches associated with regulatory actions

**Fig 4:**
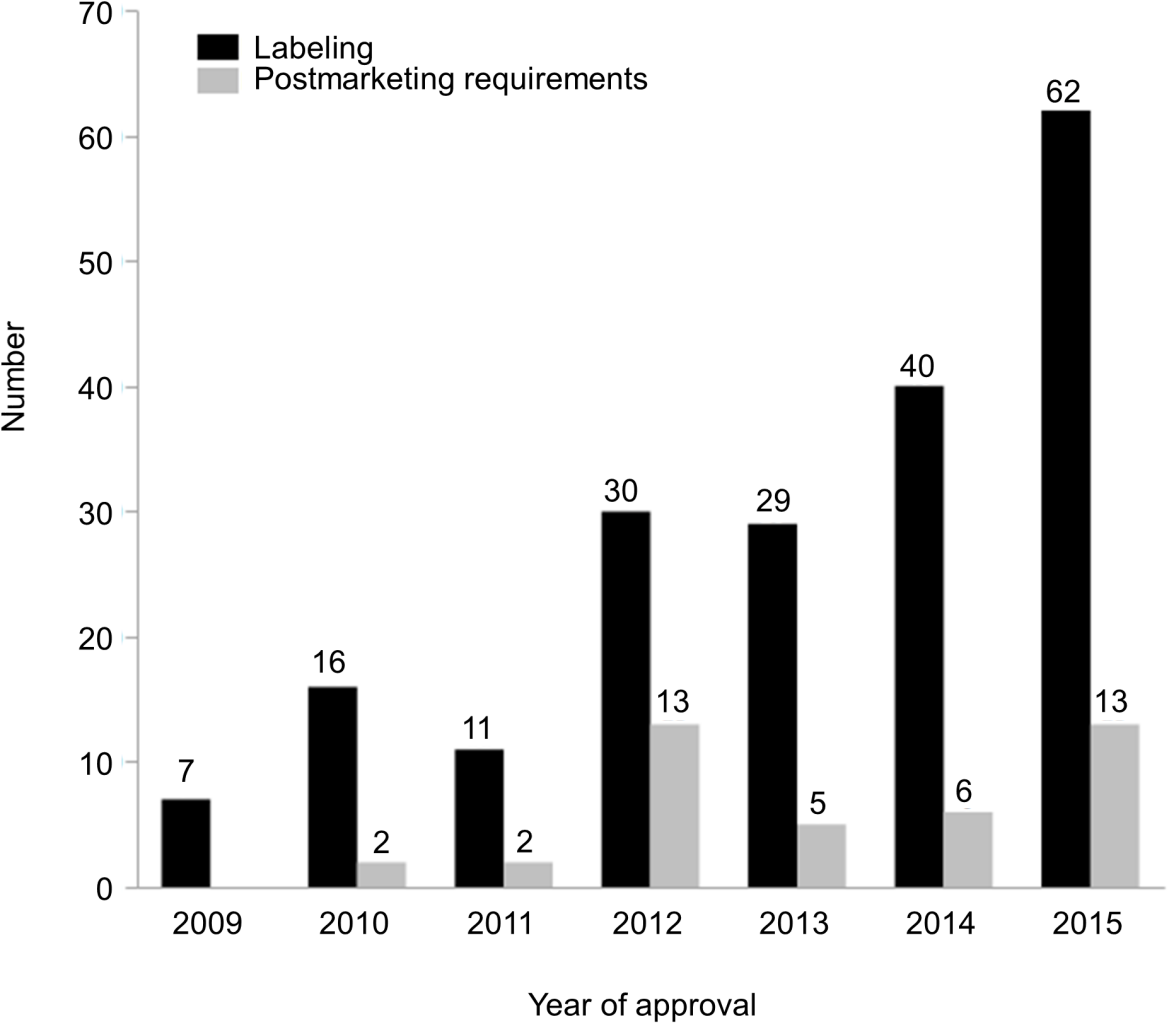
The numbers of SMQ searches which directly impacted regulatory decisions per year of approval. The regulatory decisions herein refer to those associated with labeling and postmarketing requirements. A single search may have an impact on both regulatory decisions. SMQ, Standardised MedDRA Query.

In addition to a summary of search results in the SBoAs, 227 searches (18% of searches performed) in 73 submissions (40% of the submissions investigated) seemed to have had a direct impact on regulatory decisions typically associated with labeling and/or postmarketing requirements (Table 4). The distribution of SMQ searches influencing regulatory decisions associated with labeling and postmarketing requirements by year of the NDA/BLA approval is displayed in Fig 4. If each sub-SMQ was calculated separately, the SMQs, *Severe cutaneous adverse reactions (SMQ)*, *Anaphylactic reaction (SMQ)*, *Angioedema (SMQ)*, which were often used together for the investigations of hypersensitivity were more often likely to have impact on regulatory decisions for labeling. If each sub-SMQ was not calculated separately, the SMQ *Hepatic disorders* was most likely to have impact on regulatory decisions for labeling (27 searches). Besides, searches with the SMQ *Ischaemic heart disease* were most often associated with postmarketing requirements (12 searches). Those resulting in labeling decisions most often consisted of the submissions containing antineoplastic agents (18%), based on ATC2 level classification system and those associated with postmarketing requirements most often contained the submissions of immunosuppressants (34%). Direct verification of cases retrieved and/or comparison of search results with other evidence was mentioned in 75% of the searches that seemed to have impact on regulatory actions.

**Table 4:** Characteristics of searches performed with regulatory impact.

Regulatory impact	Number of searches with direct verification of cases retrieved only (%)	Number of searches with comparison of search results with other evidence^a^ only (%)	Number of searches with the application of both approaches (%)	Number of searches without mentioning of either approach (%)	Total number of searches performed (%)^b^
Labeling^c^	43 (22)	18 (9)	83 (43)	51 (26)	195 (%)
Postmarketing Requirements^c^	7 (17)	12 (29)	18 (44)	4 (10)	41 (%)
Others^c^^,^^d^^-^^g^	1^d^ (14)	1^e^ (14)	4^f^^,^^g^ (57)	1^e^ (14)	7 (%)

^a^Such as the comparison with the report on higher Medical Dictionary for Regulatory Activities (MedDRA) levels of adverse events.

^b^Since a single search may have impact on more than one regulatory decision, the total number of searches herein exceeds 227, the number of searches performed with direct impact on regulatory decisions.

^c^Due to rounding, the percentages may not add to 100%.

^d^Medical guide/risk evaluation and mitigation strategies (REMS).

^e^Risk/benefit analysis.

^f^Pharmacovigilance plan.

^g^Tool for data safety monitoring board (DSMB).

## Discussion

Our analyses suggest that SMQs are increasingly being incorporated into safety analyses from industry and the USFDA. From 2006 to 2015, the reporting of SMQ analyses has increased in frequency by the FDA and industry, alike. Requests to perform searches were frequently found in the administrative correspondence or meeting minutes with advice for or discussion of SMQ selection, recommended search options, as well as database(s) to be searched. Senior FDA reviewers, including those at the supervisory level, frequently comment on SMQ analyses found in primary reviews. While the SMQ was used, SMQ analyses and their results have clearly become an integral part of pharmaceutical safety assessment. We have comprehensively evaluated the patterns of their usage, efforts to validate their results, and their impact on the regulatory process. From these assessments, we emphasize several tenets that are found in, or derived from, the core documents describing use of SMQs [7] and the statistical analysis of safety data [22], namely:

- Use of SMQs as a tool to screen for safety signals, rather than one that produces results upon which a diagnosis may be made
- Use of descriptive rather than inferential statistics to describe findings from SMQ analyses of clinical trials not designed for hypothesis testing
- More regularity in the documentation of SMQ modification
- Systematic verification of cases when comparisons are drawn between treatment groups or when important regulatory actions are based on the findings

Several properties of SMQs suggest their use is more appropriate for AE screening, than for conclusions that amount to a diagnosis or sophisticated statistical analysis. Collection of AE data, upon which the analyses are based, is a system not suited to quantitative analysis of or diagnosis based upon AEs without careful follow-up for those situations requiring further attention. Subjects make occasional visits to study sites, often after lengthy intervals of time, sometimes making the quality of information imprecise. Furthermore, the efforts needed to confirm potentially hundreds of SMQ-positive cases with trial data would be overwhelming to match the precision suggested by the level of statistical analysis. We therefore suggest that SMQs are best used in the screening process of data inspection. Descriptions of these data are most appropriately done with descriptive statistics than inferential testing, since the trials or post-marketing scenarios from which the AEs are derived are not optimally designed to collect these data. The long history of safety evaluators using “designated medical event” or “adverse events of special interest” term lists seemingly emboldens those using SMQs to adapt them on a case-by-case basis. A notable subset of searches applies a modified SMQ by term addition and/or exclusion, indicating an attempt at increasing the specificity or sensitivity. The MedDRA Maintenance and Support Services Organization in the SMQ introductory guide anticipates such alterations of SMQs [7].

“If any modifications are made to term content or structure of an SMQ by a subscriber or user, it can no longer be called an ‘SMQ’ but it should instead be referred to as a ‘modified MedDRA query based on an SMQ’…”

Reporting these results may become very confusing as investigators with different self-adapted versions of SMQs attempt to compare results. Verification or checking each case deemed “SMQ-positive” is a critical step in their use. Much like the allegorical fishing net described in our introduction, the broad web of search terms often ensnares cases based on terms that are non-specific but useful to ensure sensitivity in the setting of adequate corroboration. A clear example of this is *Acute pancreatitis (SMQ)*. Though a match with the AE database only requires a match on a single term, such as *nausea* [9], this is clearly insufficient to declare such a diagnosis. The verification of cases should at the least involve inspection of the adverse events from the case. Several NDAs included application materials or reviews that simply provided a table with incidences for each of the SMQs evaluated, sometimes with inferential results such as odds ratios or p-values. Beyond simply noting the associated event, SMQ specific corroborations may be used making use of specific laboratory test results, vital signs, or clusters of specific AEs. As with any clinical diagnostic analysis, confounding factors, such as medical history and concomitant medications should be considered and discussed in relation to the veracity of the findings. We were also surprised at the relatively low frequency of applications of the algorithmic capabilities for the 7 SMQs with this feature in the present study. Algorithmic SMQ-positive cases need verification because there is no requirement for a temporal association of the events for different term categories. While it may be difficult to know how proximal these events must be, it seems fairly intuitive that a rash and wheezing separated by hundreds of days are less likely to constitute anaphylaxis than if they were contemporaneous. The findings from SMQ searches supported labeling additional information and in other cases, suggested this wasn’t necessary. Similarly, SMQ search results were also used in the determination of the need, or not, for postmarketing requirements. Search results with direct regulatory impact were mostly verified and/or compared with other evidence, indicating the prudence in making regulatory decisions. The extent to which cases are investigated by the agency and industry alike should be correlated to the seriousness of the findings and magnitude of the regulatory action influenced by the finding.

Despite our comprehensive attempt at characterizing the regulatory use of SMQs, this study has limitations related primarily to the extent to which marketing applications are described in publicly available SBoAs. For example, because not all SMQ version numbers are available in review documents, it is difficult to determine which search options were available at the time of the submission. The percentage of directly verified cases or comparison with other evidence might be underestimated since every verification or comparison may not be mentioned in the application or review documentation. However, to the extent that information is publicly available, our research has systematically reviewed the use and influence of SMQs demonstrating their prevalence, usefulness, and factors critical for their successful application in safety analyses, which has not been comprehensively discussed before. Previous research focused more on the performance of SMQs [12–18]. Nonetheless, in a study dedicated to the evaluation of the performance of four SMQs for retrieval of adverse drug reactions in the French pharmacovigilance database, the importance of case-by-case analysis for excluding false positives in the analysis of *Demyelination (SMQ)* has also been pointed out although only four SMQs were investigated in the study [14].

## Conclusions

In conclusion, the present study demonstrates increasing use of SMQs in reviews of marketing applications, indicating the practicality of SMQs considered by the applicants and USFDA. SMQ search strategy depended on case-by-case considerations; however, the prevalent use of certain SMQs revealed the most common safety concerns evaluated by using SMQs. These concerns included hepatic toxicity, cardiac toxicity, central nervous system toxicity, and hypersensitivity. The more frequent use of narrow searches, when both narrow and broad terms were available, implied efforts made to enhance specificity. Although direct verification of cases retrieved was not routinely performed, SMQ searches associated with regulatory decisions were usually confirmed by direct verification of cases retrieved and/or comparison of search results with other evidence. In addition, the impact on labeling and postmarketing requirements demonstrates the utility of SMQ use. The study also presents critical suggestions for successful SMQ use as mentioned above. With appropriate choice of SMQs and search options, proper statistical analysis and thorough documentation, as well as verification for cases retrieved, the SMQ is a well-recognized tool with increasing importance for the analysis of safety profiles, which could be effectively used in making regulatory decisions. Considering their increased usage, the future trends in SMQ utilization and their regulatory implications are important issues worthy of evaluation.
